# Lack of Exercise of "Moderate to Vigorous" Intensity in People with Low Levels of Physical Activity Is a Major Discriminant for Sociodemographic Factors and Morbidity

**DOI:** 10.1371/journal.pone.0115321

**Published:** 2014-12-18

**Authors:** José A. Serrano-Sánchez, Luis M. Bello-Luján, Juan M. Auyanet-Batista, María J. Fernández-Rodríguez, Juan J. González-Henríquez

**Affiliations:** 1 Department of Physical Education and Sport Sciences, University of Las Palmas de Gran Canaria, Las Palmas de Gran Canaria, Spain; 2 Research Institute of Biomedical and Health Sciences (IUIBS), University of Las Palmas de Gran Canaria, Las Palmas de Gran Canaria, Spain; 3 Directorate General of Public Health, Canary Island Health Service, Las Palmas de Gran Canaria, Spain; 4 Department of Primary Health Care, Canary Islands Health Service, Las Palmas de Gran Canaria, Spain; 5 Department of Clinic Pathobiology, University Hospital Dr. Negrín, Las Palmas de Gran Canaria, Spain; 6 Department of Matemathics, University of Las Palmas de Gran Canaria, Las Palmas de Gran Canaria, Spain; School of Public Health, Zhejiang University, China

## Abstract

**Introduction:**

The aim is to examine the differences between participation at low and zero moderate to vigorous physical activity (MVPA) in relation to their trends and associations with known socio-demographic and health factors. We hypothesised that the number of people at zero MVPA level could be rising despite a parallel increase in the population meeting the recommended MVPA level. We also hypothesised that graded associations of sociodemographic and health factors exist across MVPA levels.

**Methods:**

Two independent population-based samples (*n = *4320 [2004] and *n = *2176 [1997]), were recruited with a stratified and random sampling procedure and interviewed at home by professional interviewers. The MVPA was assessed by validated questionnaire. The participants were classified into three MVPA levels: zero, low and recommended MVPA. The trend of each MVPA level was analysed with the standardized prevalence ratios. Correlates of low and zero MVPA levels were examined using multinomial logistic regression.

**Results:**

The population at zero and recommended MVPA levels rose between 1997–2004 by 12% (95% CI, 5–20%) and 7% (95% CI,−4–19%) respectively, while the population at low MVPA level decreased. At zero MVPA level, associative patterns were observed with sociodemographic and health factors which were different when compared to the population at low MVPA level.

**Conclusions:**

Despite the slight increase of population meeting the recommended MVPA level, a higher trend of increase was observed at zero MVPA level. Both recommended and low MPVA levels increased their participation by absorbing participants from the low MVPA level. The sociodemographic profile of those with low MVPA was more similar to the population at recommended MVPA than at zero MVPA level. Methodological implications about the combination of light and moderate-intensity PA could be derived. The prevention of decline in actual low MVPA could change the trend of increase in the population at zero MVPA level, particularly among young adults.

## Introduction

Lack of physical activity is a major health risk for premature mortality and chronic morbidity [1]–[3]. In accordance with its public importance, the promotion of physical activity has been employed as an international strategy to prevent chronic diseases, particularly cardiovascular and metabolic diseases [4]. At the present time, the minimum recommended standard of physical activity for the general adult population entails the accumulation of a total of 150 minutes per week (min/wk) of any type of physical activity at moderate or higher intensity (MVPA ≥3.5 MET) in sessions of a minimum of 10 minutes [5]–[6]. For vigorous intensity physical activity the recommendation is for at least 75 min/wk. Additional muscle-strengthening activities are also recommended to obtain health benefits [5]. For a healthy adult, the minimum recommended MVPA level is met by 30 minutes of brisk paced walking on 5 days/wk.

Historically, public health recommendations have focused on encouraging leisure time PA of at least moderate intensity and of a sufficient amount to lead to beneficial health outcomes. Participation at a level of intensity (MVPA), which induces at least a moderate increase in the respiratory rate, is important from a public health perspective. At this level of intensity an improvement in cardiorespiratory fitness is expected [7]–[8]. Cardiorespiratory fitness is one of the best predictors of longevity [1], [9] and lower morbidity [10]–[11].

Those below the recommended level of MVPA have usually been classified as inactive, sedentary or having a sedentary lifestyle [12]–[16]. However, there have been recent calls to standardize the semantic use of the term "sedentary", with suggestions that this term should be avoided when describing individuals or population and used to define behaviours ≤1.5 metabolic equivalents (METs) [17]–[18]. Instead of sedentary, inactive has been suggested as a standard term to describe individuals and population whose MVPA levels are insufficient. The rationale is that sedentary behaviour (≤1.5 METs) has been found to be associated independently of other PA types with diverse health outcomes such as obesity [19]–[20], cardio-metabolic risk [21]–[22], breast cancer [23] and mortality from all causes [24].

The classification of individuals, rather than their different physical activity behaviours, into levels of physical activity is also of interest in the field of physical activity promotion by contributing to the identification of population subgroups at health risk, and by helping to develop better tailored intervention strategies for promoting MVPA in population. The recommended MVPA level has been widely used in epidemiological research as an operational definition to classify participants for the examination of population trends [25]–[30] and sociodemographic correlates of MVPA [31]–[35]. Unlike the agreement about what constitutes sedentary behaviour, there is no established definition of what constitutes an inactive person. The most commonly used operational definition of the inactive level is below MVPA recommended level, but other operational definitions have also been employed, including the absence of MVPA [3], [36]–[37], ratio of energy expenditure in MVPA/total physical activity (i.e.<10%) [14]–[15], [38] and total physical activity energy expenditure (i.e., <1.5 Kcal/day/kg, <10 METs-hour/week) [31], [39]. Consequently, classification as inactive frequently includes those who do participate in MVPA but below the recommended level and those whose energy expenditure is exclusively in light PA and sedentary behaviours with a total absence of MVPA.

A temporal trend of increase of population meeting MVPA guidelines has been reported in USA (1998–2005) [28], Sweden (1990–2007)[40], Denmark (1987–2005)[41], England (1991–2004) [26] and Spain (1995–2003) [29]. A complementary trend of a reduction in the inactive population has been reported in USA (1994–2004)[42], Canada (1994–2005)[39] and Finland (1972–1997)[43]. In Spain, some studies have found a reduction in the inactive population (1995–2005) using a definition which classified as inactive those below the MVPA recommended level [29]. However, this reduction in the inactive population could be masking a trend of increase in population abandoning the MVPA intensity. It is plausible that the proportion of the adult population meeting the MVPA recommended level increases with a concomitant increase of the proportion stopping MVPA intensity. Correlates for the population at zero MVPA level could also be different from those for the population at low MVPA level, but the two are often combined to define inactivity. There is little information about potential differences among the inactive population (those below recommended MVPA level) regarding the intensity of physical activity performed.

The purpose of the present study is to examine potential differences in correlates and temporal trends among the inactive population considering the intensity of the leisure time physical activity performed. This entails comparisons between the population with some MVPA but below the recommended MVPA level and those population at zero MVPA level. We hypothesize that the number of people at zero MVPA level could be rising in parallel with the population meeting the recommended MVPA level. Also, the intensity of the physical activity performed could affect the consistency of graded relationships of known MVPA correlates with low and zero MVPA levels.

## Methods

### Sample and design

The data were obtained from two independent samples used in the Canary Islands Health Survey of 1997 (*n = *2176) and 2004 (*n = *4320). The adult participants were informed of the objectives and their oral consent requested. If the adult participants agreed, the interviewer was invited into their home to conduct the interview. Verbal consent was sufficient and all the interviews were recorded and analyzed anonymously. A codec-number was used to record the consent. Written consent for those under the required legal age were obtained from legal tutors present in the home. If no legal tutor was available a second visit was attempted. If no legal tutor was present in the second visit, another home was randomly selected in the same census tract. The bioethics committee of the Canary Islands Health Service approved the procedures. The surveys employed multi-stage sampling stratified by island, district, municipal size and socioeconomic level of the census tracts, with a proportional distribution by age group and sex [44]. In 2004, the number of interviews in the older female group was increased to obtain more precise results for this collective. The number of census tracts and dwellings per tract was estimated through a linear cost function and statistical precision [44]. The total number of census tracts/dwellings per tract was 180/25 (2004) and 109/20 (1997), giving a sampling error of ±1.9% (2004) and ±2.8% (1997) for estimation of the inactive population and taking into consideration the design effect [45]. Participants were interviewed at their homes by professional interviewers, who were trained in the application of the questionnaire, including specific questions of physical activity. When the selected participant was not at home another family member of the same profile and sex was interviewed in their place. If no other suitable family member was available a second visit was attempted. If a second visit was unsuccessful, the nearest available dwelling was chosen as an alternative. The data of 1997 and 2004 were acquired in the months of June, July and August.

### Assessment of physical activity and covariables

To evaluate leisure time MVPA the questions used were taken from the CINDI (Countrywide Integrated Noncommunicable Diseases Intervention Programme) survey of the World Health Organisation [46]. The questionnaire included 3 questions: **1)** How much PA do you have during your leisure-time? (If it varies with the seasons, mention the group that best represents the average of the year) (a. In my leisure time I read, watch television and do things that do not require PA; b. In my leisure time I walk, ride a bicycle or move in other ways requiring PA for at least 4 hours a week. This includes walking, fishing and hunting, lighter garden work and so on, but not going to and coming from work. c. In my leisure time I have PAs to maintain fitness, such as running, skiing, gymnastics, swimming, ball-games or doing heavy garden work or its equivalent; d. In my leisure time I train regularly, several days a week, for competitions in running, orienteering, ball-games or other physically heavy sports; **2)** How often do you do activities lasting at least 20–30 minutes that make you short of breath and perspire? (open-ended question in days per week), **3)** How long do your episodes of physical activity last? (open-ended question in minutes per day). Validation of these questions was tested using as criteria cardiorespiratory fitness (indirect VO_2_ max) and several cardiovascular risk factors in 652 adults (20–59 years old) [47]. For cardiorespiratory fitness, correlations with the questions were between 0.20–0.36, similar to those found for other international physical activity questionnaires [48]–[49]. The PA-CINDI questionnaire also showed good sensitivity to express significant differences between three levels of MVPA (low, moderate and high) in cardiorespiratory fitness, diastolic blood pleasure, total cholesterol, high density lipoprotein cholesterol and smoking. Reliability of the physical activity questions used in our study was tested on 480 participants two year later to examine whether changes in physical activities were associated to changes in several criterion measures obtained by exercise and analytical tests, showing that those participants who had increased their physical activity level expressed significant increases in maximum work load, total performed work load, high density lipoprotein cholesterol and a decrease in triglycerides, total cholesterol, systolic blood pressure, time for restoration of pulse rate and blood pressure [47]. The participants of our study were classified according to recommended MVPA levels [5]–[6], and additionally those participants below the recommended level were segregated into two groups with and without MVPA. The cut-off points for the 3 MVPA levels used for this study were: *recommended MVPA* (MVPA ≥5 days/wk and at least 30 min/day), *low MVPA* (MVPA <5 days/wk or <30 min/day and ≥1 day/week) and *zero MVPA* (no MVPA per week).

The questionnaire included additional standardised questions to obtain sociodemographic data and information concerning health behaviour and chronic morbidities: age, sex, occupation, educational level, marital status, perceived health, smoking habit and perceived fitness. Table 1 shows the categories used in the analyses. Perceived fitness involved asking the participants to provide a self-assessment on a scale of 1 to 5 (from very bad to very good). This question was shown to be a good predictor of mortality in a long-term prospective study (1988–2001) [50]. Participants were considered to be suffering from high blood pressure, diabetes, cholesterol disorders or rheumatic pain when they reported that their doctor had diagnosed them as such. The number of accumulated morbidities was also calculated for each participant (zero, one, two and three or more).

**Table 1:** Characteristics of the participants in the Canary Islands Health Survey 1997–2004.

		1997 (*n = *2176)	2004 (*n = *4320)	
		% (95% CI) d	% (95% CI) d	*p*
Sex [% (95% CI)] a				
	Female	51.9 (48.9–55.0)	57.8 (55.5–60.1)	.000
	Male	48.2 (45.3–51.2)	42.3 (40.3–44.4)	.000
Age [% (95% CI)] b				
	16–30	31.3 (29.0–33.7)	21.7 (20.3–23.1)	.000
	31–45	28.0 (25.8–30.2)	30.2 (28.5–31.9)	.093
	46–60	21.1 (19.1–23.0)	19.8 (18.4–21.1)	.217
	> 60	19.8 (17.9–21.7)	28.6 (27.1–30.2)	.000
Age (mean ± SD)		42.5±18.5	47.5±18.8	.000
Level of Education [% (95% CI)] c				
	Primary or lesser	66.5 (63.1–69.8)	67.5 (65.3–69.7)	.407
	Secondary	24.4 (22.5–26.4)	24.7 (23.2–26.2)	.793
	University	9.1 (7.8–10.3)	7.8 (7.1–8.5)	.067
Marital status [% (95% CI)] c				
	Single	30.0 (27.9–32.1)	32.5 (30.8–34.1)	.179
	Married/in partnership	58.2 (55.0–61.4)	53.3 (51.3–55.4)	.236
	Separated/Widowed	11.8 (10.1–13.5)	14.2 (13.4–15.0)	.095
Occupational status [% (95% CI)] c				
	In employment	46.3 (43.4–49.1)	47.9 (45.8–50.0)	.125
	Unemployed	8.5 (7.4–9.7)	11.6 (10.7–12.4)	.029
	Student	9.7 (8.6–10.8)	7.4 (6.7–8.1)	.000
	Home care	21.7 (19.9–23.5)	14.2 (13.4–15.0)	.000
	Pensioner, retired	13.8 (12.4–15.3)	19.0 (18.0–20.0)	.000
Perceived health [% (95% CI)] c				
	Good or very good	67.0 (63.6–70.4)	68.7 (66.2–71.2)	.163
	Fair	21.3 (19.4–23.1)	24.9 (23.7–26.0)	.001
	Bad or very bad	11.2 (9.9–12.5)	5.9 (5.5–6.3)	.000
Smoking habit [% (95%CI)] c				
	Non-smoker	65.0 (61.7–68.3)	68.6 (66.3–70.9)	.084
	<10 cig./day	7.7 (6.6–8.7)	7.7 (7.0–8.3)	.657
	10–19 cig./day	9.9 (8.6–11.1)	8.4 (7.7–9.1)	.082
	≥20 cig./day	17.5 (15.7–19.2)	15.3 (14.2–16.4)	.256
Perceived fitness [% (95% CI)] c				
	Good or very good	23.8 (21.8–25.8)	36.6 (34.8–38.3)	.000
	Normal	55.7 (52.6–58.8)	46.1 (44.3–48.0)	.000
	Bad or very bad	20.5 (18.7–22.3)	17.3 (16.3–18.3)	.000
Morbidity [% (95% CI)] c				
	Cholesterol disorders	9.9 (8.8–11.0)	11.8 (11.1–12.5)	.000
	High blood pressure	13.3 (11.9–14.8)	16.0 (15.0–16.9)	.000
	Diabetes	5.3 (4.5–6.2)	6.8 (6.3–7.4)	.000
	Rheumatic pain	20.6 (18.8–22.4)	19.5 (18.5–20.5)	.456
Number of morbidities [% (95% CI)] c				
	Zero	58.0 (54.9–61.2)	57.8 (55.5–60.0)	.379
	One	23.4 (21.5–25.4)	22.8 (21.5–24.1)	.581
	Two	12.3 (10.9–13.7)	11.2 (10.4–12.0)	.094
	Three or more	6.2 (5.3–7.2)	8.3 (7.7–8.9)	.000

a Standardised by age,

b Standardised by sex,

c Standardised by age and sex using the direct method with the Spanish population as the standard.

d (95% CI)  = 95% Confidence Interval,

* *p* values for differences between 1997–2004.

### Data analysis

To analyse the trend in each of the three MVPA levels, the standardised prevalence ratio (SPR) 2004/1997 by age and sex was used [51]. All trend analyses were standardised using the direct method, taking as the standard the age and gender structure of the Spanish population. The confidence intervals of the SPR were calculated following the procedure described by Rothman and Greenland [52].

Multinomial logistic regression was used to analyse the multivariate associations between the independent variables and the three MVPA levels. In this correlational study, the data from both surveys (1997 and 2004) were analysed jointly (*n = *6496), including the year as a confounding variable. The potential differences for leisure time MVPA between census tracts, districts and islands were tested with a multilevel analysis [53]. The variance partition coefficients for the estimations of “zero MVPA” (2.9%, *p = *0.176) and “low MVPA” (3.5%, *p = *0.086) were not significant at census tract level and fell as the level (district and island) rose. Design effect was <1.75 for zero and low MVPA levels, suggesting that a fixed effects analysis at the individual level was appropriate for the data structure [54]–[55].

The results of the multinomial logistic regression are reported in terms of odds ratios (OR), confidence interval (95% CI) and statistical significance (*p*-value). The results are presented in bivariate form and adjusted for prior covariate selection obtained by stepwise analysis. The final model was selected with significant contributions (*p<*0.05) of age, sex, survey year, educational level, smoking habit, perceived fitness and 2 morbidities (cholesterol disorders and diabetes). The perceived health variable was discarded from the final analysis due to its association with perceived fitness (*r = *0.41, *p<*0.05) and because it led to confusion in the results. In the final model, two other morbidities were included (high blood pressure and rheumatic pain) for their theoretical interest. The goodness-of-fit of the multivariate model was verified with Pearson's Chi-Square test (*p = *0.331), showing a correct fit of the model [56] with the 3 MVPA levels as dependent variable. The total percentage of correctly predicted cases was 62.5%. Data analyses were performed with the R statistical package [57] and the multinomial logistic regression module of the SPSS v.19 software package [58].

## Results

Table 1 shows the characteristics of the two samples. A slight increase can be observed in 2004 in high blood pressure, cholesterol disorders and diabetes sufferers, as well as in the number of those suffering from three or more morbidities, the unemployed, pensioners and those with perceived good fitness. The differences between the limits of the confidence intervals (95% CI) however were in general very slight. The other categories revealed no consistent differences after standardising for age and sex.

### Trend of the MVPA levels

**Figure 1:**
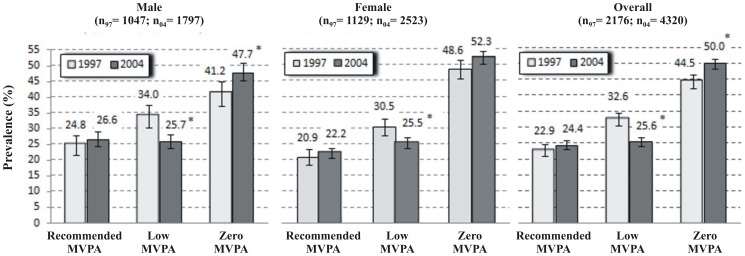
Changes in the prevalence of moderate to vigorous physical activity levels by sex. Results of prevalence were standardized by age using the direct method. The error bars represent the 95% confidence interval.

Fig. 1 shows the prevalence of the three MVPA levels analyzed in the period for males, females and overall. Zero MVPA was predominant in both samples and for both sexes (Fig. 1). In 2004, the prevalence of adults at zero MVPA level reached 50% (95% CI, 48.1–51.9%) and was higher in women than men (52.3% and 47.7% respectively, *p<*0.05). A 12% increase in the number of adults at zero MVPA level was observed (*SPR* = 1.12, 95% CI, 1.05–1.20) and the increase was higher in men vs. women (see Table 2). The number of adults at low MVPA level underwent a significant fall of 16% and 24% in women and men, respectively (*p<*0.05). The recommended MVPA level rose by 7%, though this result was not statistically significant (*SPR* = 1.07, 95% CI, 0.96–1.19, Table 2).

**Table 2:** Trend of moderate to vigorous physical activity levels according to selected characteristics.

		Recommended MVPA			Low MVPA			Zero MVPA		
		Prevalence		Trend	Prevalence		Trend	Prevalence		Trend
		1997	2004	SPR (95% CI) ^a^	1997	2004	SPR (95% CI) ^a^	1997	2004	SPR (95% CI) ^a^
All										
	%	22.9	24.4	1.07 (0.96–1.19)	32.6	25.6	0.78 (0.71–0.84) *	44.5	50.0	1.12 (1.05–1.20) *
	N	497	1021	—	707	1045	—	972	2254	—
Sex										
	Female	20.9	22.2	1.06 (0.91–1.24)	30.5	25.5	0.84 (0.73–0.95) *	48.6	52.3	1.08 (0.97–1.19)
	Male	24.8	26.6	1.07 (0.92–1.25)	34.0	25.7	0.76 (0.66–0.87) *	41.2	47.7	1.16 (1.03–1.30) *
Age										
	16–30	25.9	27.0	1.04 (0.86–1.27)	42.4	32.3	0.76 (0.65–0.89) *	31.6	40.7	1.29 (1.09–1.52) *
	31–45	24.4	24.0	0.98 (0.81–1.20)	34.8	27.5	0.79 (0.67–0.94) *	40.9	48.6	1.19 (1.03–1.38) *
	46–60	21.5	24.4	1.14 (0.89–1.45)	26.7	23.7	0.89 (0.71–1.11)	51.8	51.9	1.00 (0.85–1.17)
	>60	17.6	21.5	1.22 (0.94–1.58)	20.0	15.0	0.75 (0.58–0.97)	62.4	63.5	1.02 (0.89–1.17)
Level of education										
	Primary or lesser	22.0	23.7	1.08 (0.93–1.24)	29.0	22.2	0.76 (0.67–0.87) *	49.0	54.1	1.11 (1.01–1.21) *
	Secondary	25.4	24.9	0.98 (0.77–1.26)	36.4	32.4	0.89 (0.73–0.89) *	38.2	42.7	1.12 (0.90–1.38)
	University	28.9	31.8	1.10 (0.78–1.56)	38.7	27.6	0.71 (0.52–0.97) *	32.4	40.6	1.25 (0.92–1.71)
Marital status										
	Single	23.6	24.3	1.03 (0.80–1.32)	36.7	26.6	0.73 (0.59–0.89) *	39.7	49.1	1.24 (1.01–1.51) *
	Married/with partner	23.3	25.1	1.07 (0.92–1.26)	29.2	23.5	0.80 (0.69–0.94) *	47.5	51.4	1.08 (0.97–1.21)
	Separated/widowed	19.4	24.0	1.24 (0.66–2.32)	25.5	18.0	0.70 (0.42–1.18)	55.1	58.1	1.05 (0.76–1.47)
Occupational status										
	Student	33.1	29.3	0.89 (0.34–2.29)	49.5	30.6	0.62 (0.36–1.07)	17.4	40.1	2.30 (1.18–4.51) *
	Unemployed	22.0	24.4	1.11 (0.62–1.96)	36.4	25.5	0.70 (0.51–0.97) *	41.6	50.1	1.21 (0.84–1.73)
	Employed	23.9	23.6	0.99 (0.83–1.18)	34.3	26.6	0.77 (0.66–0.91) *	41.8	49.8	1.19 (1.04–1.37) *
	Home care	22.9	23.3	1.02 (0.76–1.37)	25.2	21.7	0.86 (0.65–1.15)	51.9	55.0	1.06 (0.88–1.27)
	Retired	9.5	24.5	2.58 (1.61–4.13) *	16.2	21.9	1.35 (0.77–2.37)	74.2	53.6	0.72 (0.55–0.95) *
Perceived health										
	Good-very good	25.6	27.1	1.06 (0.94–1.20)	34.6	27.6	0.80 (0.71–0.89) *	39.8	45.2	1.14 (1.03–1.26) *
	Fair	19.9	19.6	0.98 (0.73–1.32)	30.4	20.2	0.67 (0.52–0.86) *	49.7	60.2	1.21 (1.03–1.43) *
	Bad-very bad	13.5	10.1	0.75 (0.37–1.51)	16.1	22.9	1.42 (0.77–2.63)	70.4	67.0	0.95 (0.71–1.28)
Smoking habit										
	Non-smoker	24.0	25.2	1.05 (0.92–1.21)	33.3	27.5	0.83 (0.73–0.94) *	42.8	47.3	1.11 (1.00–1.22) *
	<10 cig/day	26.5	24.7	0.93 (0.61–1.42)	31.6	29.3	0.93 (0.65–1.32)	41.8	46.0	1.10 (0.79–1.52)
	10–19 cig/day	27.8	29.2	1.05 (0.72–1.52)	38.4	24.2	0.63 (0.44–0.89) *	33.8	46.6	1.16 (0.95–1.41)
	≥20 cig/day	17.6	17.2	0.98 (0.67–1.42)	26.5	18.1	0.68 (0.50–0.94) *	55.9	64.7	1.38 (1.02–1.87) *
Perceived fitness										
	Good-very good	37.1	34.0	0.92 (0.77–1.09)	35.4	29.7	0.84 (0.70–1.00)	27.4	36.2	1.32 (1.08–1.61) *
	Normal	21.7	22.6	1.04 (0.89–1.22)	34.4	25.0	0.73 (0.63–0.83) *	43.9	52.4	1.20 (1.07–1.33) *
	Bad-very bad	10.5	9.4	0.89 (0.57–1.38)	21.5	18.4	0.86 (0.62–1.18)	67.9	72.2	1.06 (0.91–1.24)
Chronic Morbidity										
	Cholesterol disorders	14.3	16.5	1.15 (0.67–1.97)	41.8	26.7	0.64 (0.37–1.11)	43.9	56.8	1.29 (0.98–1.72)
	High blood pressure	19.7	20.1	1.02 (0.63–1.65)	27.1	21.5	0.79 (0.49–1.27)	53.2	58.3	1.10 (0.82–1.47)
	Diabetes	25.4	15.2	0.60 (0.19–1.89)	17.1	21.0	1.23 (0.53–2.84)	57.5	63.8	1.11 (0.62–2.00)
	Rheumatic pain	20.3	16.5	0.82 (0.54–1.24)	29.6	25.1	0.85 (0.56–1.29)	50.1	58.3	1.16 (0.94–1.44)
Number of morbidities										
	Zero	24.2	27.3	1.13 (0.97–1.30)	31.6	26.1	0.83 (0.73–0.94) *	44.2	46.6	1.05 (0.94–1.18)
	One	24.5	22.8	0.93 (0.73–1.18)	33.5	27.7	0.83 (0.67–1.02)	42.0	49.5	1.18 (1.02–1.39) *
	Two	23.0	17.1	0.74 (0.46–1.21)	25.2	23.9	0.95 (0.60–1.51)	51.8	59.0	1.14 (0.87–1.49)
	Three or more	19.2	14.2	0.74 (0.20–2.77)	21.4	24.4	1.14 (0.60–2.15)	59.4	61.4	1.03 (0.63–1.70)

### Trend of the MVPA levels by sociodemographic group and health factors

Table 2 shows the prevalence and trend of the three MVPA levels analyzed. Sixteen of the 36 sociodemographic and health categories examined at zero MVPA level revealed significant changes (Table 2), most of them as trends of increase particularly in men, students, 16–30 group, good perceived fitness, single and heavy smokers (≥ 20 cigarettes/day). Only the retired category showed a significant reduction in participation at zero MVPA level. A complementary trend to that observed for the population at zero MVPA level was observed at low MVPA level. Nineteen sociodemographic and health groups showed a significant reduction in participation at that level (*SPR* between 0.63 and 0.83, *p<*0.05, Table 2). At the recommended MVPA level, only one category (retired) revealed a significant trend of increase.

### Associations of the sociodemographic and health factors with the MVPA levels

Table 3 shows the results of the multinomial logistic regression analyses for zero and low MVPA levels vs. recommended MVPA levels as reference. The rise in age and fall in level of education and perceived fitness were independently associated with a higher prevalence at zero vs. recommended MVPA level. In addition, women, heavy smokers, those who reported cholesterol disorders or diabetes and those with three or more chronic conditions showed a higher probability of zero MVPA in their leisure time.

**Table 3:** Associations between levels of physical activity and selected sociodemographic and health characteristics.

		Zero MVPA (*n = *3226)				Low MVPA (*n = *1752)			
		Raw		Adjusted a		Raw		Adjusted a	
		OR^b^	(95%CI)	OR^b^	(95%CI)	OR^b^	(95%CI)	OR^b^	(95% CI)
Year									
	1997	1		1		1		1	
	2004	**1.13**	**(1.03–1.25)** *	**1.28**	**(1.11–1.47)** *	**0.72**	**(0.62–0.83)** *	**0.78**	**(0.67–0.91)** *
Sex									
	Male	1		1		1		1	
	Female	**1.38**	**(1.22–1.56)** *	**1.20**	**(1.05–1.37)** *	1.13	(0.98–1.29)	1.07	(0.92–1.23)
Age									
	16–30	1		1		1		1	
	31–45	**1.37**	**(1.16–1.62)** *	1.15	(0.94–1.41)	0.90	(0.75–1.07)	0.89	(0.74–1.06)
	46–60	**1.61**	**(1.34–1.93)** *	**1.19**	**(1.04–1.42)** *	**0.78**	**(0.64–0.96)** *	**0.75**	**(0.61–0.93)** *
	>60	**2.26**	**(1.89–2.69)** *	**1.33**	**(1.08–1.65)** *	**0.59**	**(0.48–0.72)** *	**0.54**	**(0.43–0.69)** *
Level of education									
	University	1		1		1		1	
	Secondary	1.27	(0.99–1.62)	**1.39**	**(1.08–1.80)** *	**1.32**	**(1.03–1.70)** *	1.27	(0.99–1.64)
	Primary or lesser	**2.05**	**(1.64–2.56)** *	**1.62**	**(1.28–2.05)** *	0.93	(0.73–1.17)	0.95	(0.75–1.20)
Smoking habit									
	Non smoker	1		1		1		1	
	<10 cig/day	0.74	(0.60–1.05)	0.97	(0.77–1.22)	1.04	(0.81–1.33)	1.01	(0.78–1.31)
	10–19 cig/day	0.80	(0.63–1.01)	0.99	(0.77–1.26)	0.93	(0.73–1.18)	0.88	(0.69–1.13)
	≥20 cig/day	**1.56**	**(1.30–1.87)** *	**1.92**	**(1.57–2.33)** *	1.06	(0.85–1.31)	1.03	(0.83–1.29)
Perceived fitness									
	Very good, good	1		1		1		1	
	Normal	**2.41**	**(2.10–2.76)** *	**2.23**	**(1.93–2.56)** *	**1.40**	**(1.21–1.62)** *	**1.45**	**(1.25–1.69)** *
	Bad, very bad	**8.78**	**(7.04–10.93)** *	**7.24**	**(5.76–9.09)** *	**1.94**	**(1.51–2.50)** *	**2.20**	**(1.70–2.86)** *
Morbidity									
	Zero	1		1		1		1	
	Cholesterol disorders	**1.92**	**(1.56–2.36)** *	**1.45**	**(1.12–1.89)** *	1.23	(0.97–1.56)	1.19	(0.95–1.51)
	High blood pressure	**1.63**	**(1.38–1.93)** *	0.91	(0.75–1.11)	0.79	(0.64–1.03)	0.79	(0.63–1.00)
	Diabetes	**2.37**	**(1.81–3.10)** *	**1.49**	**(1.11–2.01)** *	1.14	(0.83–1.58)	1.37	(0.97–1.94)
	Rheumatic pain	**2.05**	**(1.75–2.39)** *	1.18	(0.99–1.42)	0.98	(0.82–1.19)	1.13	(0.91–1.39)
Number of morbidities									
	Zero	1		1		1		1	
	One	1.27	**(1.09–1.48)** *	0.99	(0.84–1.17)	1.05	(0.89–1.24)	1.16	(0.97–1.38)
	Two	1.92	**(1.58–2.34)** *	1.18	(0.99–1.42)	0.92	(0.73–1.17)	1.07	(0.83–1.38)
	Three or more	3.19	**(2.48–4.11)** *	**1.69**	**(1.27–2.24)** *	1.10	(0.81–1.50)	**1.39**	**(1.05–1.94)** *

Note: the reference category is the recommended MVPA level (*n = *1518).

a Adjusted for survey year, age, sex, educational level, perceived fitness and the four morbidities or alternatively the number of morbidities.

b OR  =  odds ratio by multinomial logistic regression.

* *p<*0.05.

At low MVPA level, only perceived fitness and three or more morbidities continued to have the direct associations seen at zero MVPA level (Table 3). Of the remaining variables and categories, year and age showed an association with low MVPA that was the opposite of that seen with zero MVPA, while the associations for women, educational level, smoking, cholesterol disorders and diabetes were not observed at low MVPA level.

## Discussion

This study was designed with the aim of examining differences among the inactive population at low and zero MVPA levels in relation with their trends and associations with sociodemographic factors, perceived physical fitness and some chronic morbidities. With respect to trends, the results showed that participation at zero MVPA and recommended MVPA levels rose over the study period whilst participation at low MVPA level decreased. The shift from the low MVPA level was mainly in the direction of zero MVPA and somewhat less in the direction of recommended MVPA level which was not significant. This trend suggests that monitoring the transition from low to zero MVPA level is a potential prevention strategy due to its capacity to reduce the numbers of those dropping out of the MVPA intensity and to increase the population at recommended MVPA level. Small increments in frequency and duration of MVPA among the population with low MVPA would increase the population at recommended MVPA level and reduce the population at zero MVPA level.

The intensity of the physical activity is important to obtain health benefits because at moderate or higher levels it activates relevant molecular mechanisms in the oxidation of fatty acids and the transport of glucose to the interior of the muscle fibre [59]–[61]. Some of these mechanisms such as the activity of AMP-activated protein kinase may be altered in obese and diabetic patients [62]–[63], so moderate physical activity intensity could play a relevant role in the prevention of these chronic conditions. The monitoring of changes in population physical activity intensity is of interest in the field of physical activity promotion to obtain better results in interventions.

If MVPA intensity is contraindicated, the accumulation of time in light physical activity is a valid alternative to prevent the risk of inflammation in older adults [64] and improve their quality of life and physical health [65]. In healthy middle-aged adults, light physical activity measured by accelerometer has also been associated with an improvement in the 2-hr plasma glucose test [66]. In contrast, other longitudinal studies using questionnaires to assess physical activity have not found associations of light physical activity with a 10-year Framingham risk score [67] nor with the risk of mortality due to cardiovascular diseases, coronary heart disease or any other cause of mortality [68]. The dose of light physical activity for health benefits in the general population remains unclear. Light physical activity is seen as an alternative to moderate and higher intensity for special groups (e.g., dependents, older adults) and to mitigate the negative effect of sedentary behaviour on health in the general population [66], [69]. However, there are no specific standardised recommendations of how much light physical activity is good for health. The best option for general health improvement is to perform 150 min/week of moderate or higher-intensity activities [5] and reduce time in sedentary pursuits [70], including breaks in sedentary time [71].

The temporal trend of increase at zero MVPA level in our study was particularly observed in students and younger participants. It is coherent with the temporal trend observed in the population of Madrid (1995–2008) [36], and could be indicative of a change in young people's lifestyle. The decrease in walking, which is the most prevalent physical activity, has been proposed as an explanation of the rise in population with zero MVPA [36], but other explanations for younger people have been suggested including the rise in time given to sedentary occupations [72]–[74], the increase in academic pressure [75] and out-of-school study time [76], and the accumulation of time in front of several screens [77].

In our study, 3 out of every 4 participants in 2004 were below the recommended MVPA level. This is in agreement with other European studies on the adult Spanish population which have reported corresponding values of between 68 and 74% [15], [78]–[79]. There exists strong evidence of a relationship of MVPA (negative) and of the accumulation of sitting time (positive) with the group of risk factors that comprise metabolic syndrome [80]–[81]. The low levels of MVPA in the Canary Islands could explain the high rate of metabolic syndrome found there [82], in fact we found an independent risk of having three or more chronic conditions for the population with zero MVPA.

The associations of low and zero MVPA levels with the sociodemographic and health variables were quite different when compared against the same reference (the recommended MVPA level). We observed that participation at zero MVPA level rose with age and survey year whereas participation at low MVPA level showed the opposite associations. In addition, sex, educational level, smoking habit and being diabetic displayed independent associations with zero MVPA but no association with low MVPA. The only two characteristics which showed significant associations in the same direction with both MVPA levels were perceived fitness and having 3 or more morbidities. This was contrary to what we expected for a graded association of the analyzed correlates across the 3 MVPA levels. In contrast, the profile of the population with low MVPA was more similar to that at the recommended MVPA level rather than to the population with zero MVPA, with the exceptions being for perceived fitness and the accumulation of 3 or more morbidities. This suggests that the intensity of PA performed among those at zero or low MVPA level tends to produce more qualitative or class differences instead of graded relationships. Practical implications in the operational definition of inactive could be derived because the low MVPA group could introduce noise in the associations with sociodemographic and health factors when it is combined together with the zero MVPA group to define the inactive category.

The absence of MVPA was significantly higher in those who reported cholesterol disorders, diabetes or having 3 or more chronic morbidities after adjusting for principal covariates. Bearing in mind these medical conditions had been diagnosed and prescribed for, these results would suggest the need for greater emphasis on prescribing MVPA in the health care of the chronically ill because those who are in most need of attaining the recommended MVPA level for health reasons are precisely those who are most strongly associated with a lifestyle absent of MVPA.

The present study has a number of limitations and strengths. The questionnaire as a data collection system is less precise than other objective methods, e.g., accelerometers, in terms of MVPA measurement. However, it is the most cost-effective method for the assessment of physical activity in large populations, enabling the estimation of patterns and trends with moderate validity and good reliability [83]-[84]. Grouping by levels helped to mitigate questionnaire MVPA overestimation [85], reducing classification errors. Another limitation is that cross-sectional studies do not allow the establishment of causality relationships, something that could be achieved with longitudinal or intervention designs. The prevalence of chronic morbidities in our study was an underestimation of actual prevalence [86]–[87], as it only evaluated morbidities known by participants aged 16 and over, with the focus being the MVPA levels of those whose medical condition had been diagnosed. One of the strengths of the study was the use of the same questions in the two surveys enabling control of one of the principal sources of variability in trend studies [88]. Also, the standardisation carried out using the national population as the standard facilitated its comparison with other studies that have been undertaken of the Spanish population. The stability of the weather conditions in the Canary Islands (year round temperatures of 18–24°C, 21 days of rain per year and 65–70% ambient humidity) ensured control of this potential source of variability, particularly in the physical activity of walking [89] which is the main contributor in recommended MVPA levels at population level [36], [90].

## Conclusions

Differences were observed between the temporal trends and correlates of the population at zero and low MVPA levels. An increase was observed over the study period in the population at zero MVPA level by a mechanism of transference from low MVPA level. Students and younger groups showed the greatest increase at zero MVPA level. The combination of zero and low MVPA in the same category, to define the inactive group, could be concealing the actual temporal trend of the population at zero MVPA level.

Zero and low MVPA also showed great differences for almost all examined correlates. Those with zero MVPA showed independent associations with age, sex, education, perceived fitness, heavy smokers, cholesterol disorders, diabetes and 3 or more morbidities, whilst those with low MVPA expressed opposite associations for age and no associations for sex, education, smoking and all separate chronic morbidities. Perceived fitness and 3 or more morbidities showed consistent and graded associations with the MVPA levels examined. The sociodemographic profile of those at low MVPA level was more similar to the recommended MVPA than the zero MVPA level. Methodological implications about the combination of PAs at different levels of intensity could be derived for separate verification of differences before combining light and moderate PA in epidemiological studies. Since the population at zero MVPA has a higher risk for some morbidities, it may be useful to identify those who might be at risk of decreasing PA or PA intensity to implement policies promoting PA for this specific sector of the population.
